# Parental costs for in-patient neonatal services for perinatal asphyxia and low birth weight in Ghana

**DOI:** 10.1371/journal.pone.0204410

**Published:** 2018-10-12

**Authors:** Christabel C. Enweronu-Laryea, Hilary D. Andoh, Audrey Frimpong-Barfi, Francis M. Asenso-Boadi

**Affiliations:** 1 Department of Child Health, University of Ghana School of Medicine and Dentistry, Accra, Ghana; 2 Greater Accra Regional Hospital, Ghana Health Service, Accra, Ghana; 3 Tema General Hospital, Ghana Health Service, Tema, Greater Accra Region, Ghana; 4 Department of Economics, University of Cape Coast, Cape Coast, Ghana; 5 Head Office, National Health Insurance Authority, Accra, Ghana; Johns Hopkins School of Public Health, UNITED STATES

## Abstract

The major causes of newborn deaths in sub-Saharan Africa are well-known and countries are gradually implementing evidence-based interventions and strategies to reduce these deaths. Facility-based care provides the best outcome for sick and or small babies; however, little is known about the cost and burden of hospital-based neonatal services on parents in West Africa, the sub-region with the highest global neonatal death burden. To estimate the actual costs borne by parents of newborns hospitalised with birth-associated brain injury (perinatal asphyxia) and preterm/low birth weight, this study examined economic costs using micro-costing bottom-up approach in two referral hospitals operating under the nationwide social health insurance scheme in an urban setting in Ghana. We prospectively assessed the process of care and parental economic costs for 25 out of 159 cases of perinatal asphyxia and 33 out of 337 cases of preterm/low birth weight admitted to hospital on the day of birth over a 3 month period. Results showed that medical-related costs accounted for 66.1% (IQR 49% - 81%) of out-of-pocket payments irrespective of health insurance status. On average, families spent 8.1% and 9.1% of their annual income on acute care for preterm/LBW and perinatal asphyxia respectively. The mean out-of-pocket expenditure for preterm/LBW was $147.6 (median $101.8) and for perinatal asphyxia was $132.3 (median $124). The study revealed important gaps in the financing and organization of health service delivery that may impact the quality of care for hospitalised newborns. It also provides information for reviewing complementary health financing options for newborn services and further economic evaluations.

## Introduction

Efforts to improve child health indices in sub-Saharan Africa have focused on the major direct causes of neonatal deaths including, perinatal asphyxia, preterm /low birth weight (LBW) and infections [1]. Available life-saving cost-effective interventions can prevent many deaths and facility-based interventions offer the best outcome to mothers and newborns [2–6]. However, health system gaps in low- and middle- income countries (LMIC) undermine effective delivery of quality facility-based neonatal care and the burden of inpatient newborn services on families has received little attention globally [7, 8].

In Ghana, newborn deaths account for over 65% and 40% of infant and under 5 deaths respectively, and birth asphyxia and complications of preterm birth, the major causes of neonatal mortality, are among the top ten causes of all national deaths [9–11]. Neonatal infections cause significant morbidity but most deaths from infections in hospitalised newborns occur in the preterm/LBW. Like other LMIC, physical access and financial constraints are major barriers to facility-based neonatal care in Ghana despite a nationwide social health insurance scheme with a fee for service (FFS) payment mechanism for medicines [12].

In 2008, the government of Ghana instituted free antenatal and postnatal services for mothers and newborns. Subsequent evaluations of the policy showed improved access to facility-based maternal services, maternal health outcomes and parental healthcare seeking behaviour but no impact on child health outcomes [13–16]. Lambon-Quayefio *et al*, with more robust data from Ghana Demographic and Health Survey 2014, found that nationwide health insurance scheme (NHIS) made neonatal services more affordable to the population, but women in urban areas with valid health insurance had significant increased risk of neonatal death irrespective of maternal education and wealth status [17]. They attributed the situation in urban areas to overstretched health facilities and substandard neonatal services.

Ghana launched a 5-years Newborn Health Strategy and Action Plan in 2014 [14]. Combined with the NHIS and free maternal-neonatal postnatal services, this strategy is predicted to significantly reduce neonatal mortality [16, 17]. We did not find any published work that prospectively examined the cost of providing facility-based in-patient neonatal services in Ghana. This study evaluated the costs health providers and families of hospitalised newborns encounter by examining the process of care to determine the best estimate of actual cost of all activities in the full cycle of care for the 2 major causes of newborn deaths at a district and a regional hospital in an urban setting in Ghana. Analysing the process and cost of healthcare services provides an opportunity to examine the utilization of resources, and assess the quality and value–outcomes achieved per dollar spent [18, 19]. This paper presents the parental direct and indirect costs of illness.

## Materials and methods

Using a cross-sectional longitudinal design and simple random sampling, eligible newborns (Table 1) were recruited within 24 hours after birth at 2 newborn referral hospitals in Greater Accra Region during May to July 2016. Bottom-up micro-costing approach based on the Kaplan and Anderson Time-Driven Activity Based Costing (TDABC) method [20] was used to collect clinical data and a patient resource-use measurement tool adapted from Thompson *et al* [21] was used to collect parental cost and activity data. TDABC is linked to value-based healthcare agenda; it prioritizes accuracy over precision and requires two key parameters, the capacity cost rate and time used to perform healthcare activities [20]. The economic categories to be considered in the evaluation of caregiver burden in healthcare services were applied [21, 22]. All activities in the full cycle of care from admission to discharge or death were collected and analysed. The study was conducted according the principles expressed in the Declaration of Helsinki after a full review and approval by the Ghana Health Service Ethics Review Committee (Study approval ID: GHS-ERC 77/02/16).

**Table 1:** Inclusion criteria for enrolment in costing neonatal care in Ghana.

Admitted at study site neonatal unit within 24 hours after birth	
Mother alive and reachable in person or by phone	
Father (or relative responsible for mother and baby) reachable in person or by phone	
No obvious congenital abnormality	
Written informed consent given by parent(s)	
**BIRTH ASPHYXIA—criteria**	
Maturity criteria:	Gestational age of 37 completed weeks and above
Weight criteria:	Birth weight 2500–3999 grams
Evidence of foetal distress:	Abnormal cardiotocography or partograph
	Required bag and mask resuscitation at birth
Evidence of neurological deficit:	Weak or absent cry at birth
	Weak or absent suck
	Abnormal muscle tone
	Seizures
**PRETERM BIRTH / LOW BIRTH WEIGHT (LBW)- criteria**	
Maturity criteria;	Gestational age < 37 weeks
Weight criteria (categorized):	LBW: Birth weight 1500–2499 grams
	Very LBW (VLBW): Birth weight < 1500 grams

### Study sites

Ghana Health Service is the major provider of healthcare services nationwide and normal births (uncomplicated term vaginal delivery and 24 hours of postnatal observation at a health facility) were free at the point of care during the period of the study. Ghana has 10 administrative regions and each region has one referral regional hospital, several district hospitals and primary care facilities under the Regional Health Directorate. The 2 study sites were in metropolitan areas of Greater Accra Region and administrative permission was given by the Regional Health Directorate and the study sites.

The study sites, the regional hospital (RH) and largest district hospital (DH) provide inpatient services for about 40% of newborns in the region. Each hospital attends to over 9,000 births and each neonatal unit admits about 1500 newborns yearly. RH and DH were established in the precolonial era, had limited space for maternal-newborn services and provided similar level of neonatal care (including intravenous infusions, oxygen therapy, parenteral medicines, neonatal resuscitation and gavage feeding) for a similar population of newborns.

The neonatal unit at RH had 20 beds, 9 doctors, 15 certified nurses and 6 auxiliary nurses; there were 30–35 babies on admission daily and cost of oxygen therapy was paid out-of-pocket (OOP) by parents. DH had 37 beds, 6 doctors, 7 certified nurses and 7 auxiliary nurses; there were 27–39 babies on admission daily and the unit had an oxygen concentrator and parents only paid for extra oxygen therapy occasionally. Although both hospitals had radiological services it was not available at the point of care and most logistics required for laboratory services including those covered by the NHIS were dependent on the physical presence of parents.

### Study procedures

Following ethical approval a meeting was held with the paediatrician in charge of each newborn unit to discuss and share the study documents. Over a period of 2 weeks the paediatrician familiarized herself with the documents and shared the information with nurses and doctors in the newborn unit. Thereafter, researchers met with the newborn team of each hospital to understand and harmonize the care delivery value chain [19] and process of service delivery, discuss the protocol and data collection tools, plan the pre-study pilot, and identify lead nurses and doctors who will ensure 24/7 hourly accurate data entry. The protocol and study tools were slightly revised following a 2 week pre-study pilot on 10 babies. There was no interference with the organization and practice of clinical services.

To ensure consistency and avoid double counting of parental expenditure, a designated nurse (per study site) interviewed parents and supported them to complete the economic cost analysis tool. The main components of the patient resource-use measurement tool are summarized in Table 2 (S1 Appendix). Standard care in the neonatal units require mothers to visit every 3 hours (from 09.00–18.00 hours) to breastfeed and care for their babies. Fathers had separate visiting times because mothers breastfeed in the open ward. To prevent recall bias, the designated nurse contacted the newborn’s father (or mother’s designated family member) and mother (after she is discharged from hospital) in person at the hospital or by phone daily, and collected relevant data concerning their newborn’s hospitalization e.g. receipts for out-of-pocket payments, transportation costs.

**Table 2:** Domains of economic costs in patient resource-use measurement tool.

DOMAIN	CONTENT	
Socio-economic demographic data	Both parents	
Direct costs	Medical related (out-of-pocket payments)	Medicines, other therapeutics, devices, hospital stay, diagnostics/laboratory tests, clinical supplies
	Non-medical related (out-of-pocket payments)	Parental visit transportation costs, hospital accommodation costs for mothers after discharge from obstetric ward, childcare for children at home,
Indirect costs	Productivity loss	Fathers or other relevant family member - Income loss, missed working days
	Time losses (opportunity cost)	Transportation time to and fro hospital, leisure time, hospital waiting time, time spent in other hospital activities
Intangible costs	Any other way newborn’s hospitalization has affected parents/family	

To identify resource inputs and measure resource utilization a 24 hour clinical care tool that described all healthcare activities from admission to discharge was applied. As parents did not always accompany newborns at the time of admission we applied the 24 hour clinical care tool on every eligible newborn at admission and only enrolled those whose parents’ subsequently gave written informed consent within 48 hours of admission. Data on all parental activities relevant to the clinical care of newborn participants including the referral process was collected from the time of birth until discharge or death.

Valuation of resource inputs was based on resource utilization (e.g. time parents spent on medical-related hospital activities), actual OOP payments for services and products used in clinical care, and productivity losses for fathers or other relevant family member when fathers are not available. Maternal time and productivity losses were not measured because traditionally mothers stay at home for at least one month to nurse the newborn and women in employment are statutorily entitled to 3 months paid maternity leave. Mothers’ productivity losses due to preterm birth was not assessed as the objective of the study focused on cost analysis of in-patient neonatal services. Maternal transportation costs incurred for daily hospital visits to see her baby were measured and analysed. For quality assurance, parental activity and expenditure data were regularly crosschecked with the 24 hour clinical care tool and hospital medical and nursing records.

### Data analysis and cost calculations

Data were entered into excel (Microsoft 2010) database and analysed using excel functions. Qualitative data are described. Missing data on parental income were completely at random; median and mean imputations were used in the analysis [23]. The value of resources utilized in direct medical and direct non-medical healthcare were based on actual OOP payments by parents. Median time calculations were based on the time spent on activity per day when fathers visited their hospitalised newborn. Traditionally, families provide special dishes to mothers after delivery and hospitals did not offer this service; the food for mothers was not costed but the transportation cost of bringing the food from home was included in the analysis. Transportation costs for hospital birth were excluded as mothers would have accrued this cost irrespective of the newborn’s health status. For families with cars, mileage was determined with Google Maps and cost was based on government of Ghana reimbursement rate per mile in 2016 (1.07 cedis equivalent to 0.27 United States dollar). Indirect costs were valued as opportunity cost as the objective of the study was on financial constraints at the point of in-patient care. The minimum daily wage in Ghana during the study in 2016 was 8 cedis (2.05 dollars). Currency conversion was based on cedi–dollar rates during the conduct of the study (1 dollar = 3.95 cedis). All costs are reported in United States dollars.

## Results

Overall, 869 newborns were hospitalized in both hospitals during the 3 months of the study and 496 (preterm/LBW 337, perinatal asphyxia 159) newborns were eligible. Clinical data were collected from 62 babies of 58 mothers, two families gave insufficient data and were excluded from analysis. Of the 56 mothers, 55 had singleton babies and one had a set of preterm triplets; and 58 newborns (preterm/LBW 33, perinatal asphyxia 25) were recruited for the study. Six parents did not provide income data, one family was extremely poor and had no income, 94.6% (53 mothers) had valid NHIS cover, and 25 families (27 babies) had their baby admitted at RH and 31 at DH. None of the families reported having private health insurance. No case required surgical care. Five babies died and all the deaths occurred in the first week. The characteristics of families is shown in Table 3.

**Table 3:** Characteristics of families with hospitalised newborns (n = 56).

Marital status of mothers	Married		49
	Single		6
	Not specified		1
Parents’ monthly income in United States dollars	Less than 60		2
	60–150		22
	151–250		11
	251–350		9
	More than 350		5
	No data (plus one extremely poor)		7
Own car	Yes		8
	No		48
Educational status of parents		Father	Mother
	None	1	1
	Basic (6–9 years)	18	25
	Secondary (12 years)	17	8
	Tertiary	6	3
	Not specified	14	19
Employment status of parents (father/family member responsible for mother and baby)*	Full- time	37	26
	Part-time	5	4
	Unemployed	0	4
	Not specified	14	22

* Two out of 56 mothers had a family member (not father) responsible for mother and baby.

There were 25 cases of birth asphyxia (RH = 15; DH = 10) and 31 cases (families) with preterm/LBW (RH = 10; DH = 21). Among the preterm/LBW group, 54.8% (17/31) were very low birth weight (VLBW; less than 1500 grams) and 12 of the 17 VLBW babies were hospitalised at DH. Length of stay (LOS) varied from 2–41 days [median 10 days; interquartile range (IQR) 5–16 days]. The mean OOP expenditure for perinatal asphyxia was $132.3 (median $124) and for preterm/LBW was $147.6 (median $101.8).

### Direct costs

The total OOP payments varied from $27 to $332 but it cost the family with triplets $1456. Overall, total OOP payments were higher at RH than at DH (Table 4). It was statistically significantly higher for preterm/LBW non-medical related costs (*p =* 0.032) and medical related costs (*p* = 0.036), and non-medical related cost for perinatal asphyxia (p = 0.002). There was no statistical difference in medical related costs for perinatal asphyxia (*p* = 0.11). The average LOS was longer at RH, families paid a flat rate of $19 for oxygen therapy at RH but oxygen therapy was at no cost to parents at DH, and newborns at RH were more likely to be prescribed medicines and bedside diagnostics (e.g. random blood sugar measurement) that were not covered by NHIS than those at DH.

**Table 4:** Length of stay and direct costs (US dollars) at regional and district levels of care.

	Perinatal asphyxia		Preterm/Low birth weight	
Level of hospital	Regional	District	Regional	District
**Admissions May–July 2016**	81	97	149	198
**Participants (% admissions)**	15 (18.5)	10 (10.3)	12* (8.1)	21 (10.6)
**Length of stay**				
Average length of stay (LOS)	10	6	19	14
Infants with LOS 0–7 days	7	7	1	8
LOS 8–14 days	6	3	2	7
LOS > 14 days	2	0	7	6
**Non-medical related costs ($)**				
Median (IQR)	89 (45–125)	9 (3–57)	51.5 (25–113)	17 (9–37)
Mean (standard deviation)	95.4 (59.5)	26.8 (30.4)	134.9 (222)	26.6 (26.4)
Proportion of total expenditure	55%	32%	42%	35%
**Medical related costs ($)**				
Median (IQR)	71 (55–89)	43.5 (34–79)	95.5 (77–106)	53 (45–89)
Mean (standard deviation)	77.1 (28.8)	55.9 (35.3)	140.8 (154.8)	65.5 (28.2)
Proportion of total expenditure	45%	68%	58%	65%
**Outcome (number)**				
Deaths	1	0	1	3

*One family had a set of triplets.

Medical-related costs including diagnostics, medicines, and basic clinical supplies (Table 5) accounted for 66.1% (IQR 49% - 81%) of OOP payments irrespective of health insurance status. Of the 56 families, 33 newborns had blood tests covered by NHIS, 54 were given antibiotics but only 13 had blood culture tests. Seventeen families (9 in RH, 8 in DH) payed out-of-pocket for laboratory services not covered by NHIS, comprising 25% (6/24) of parents earning less than $150 monthly and 36% (9/25) earning over $150, two families did not provide income data. The nurses and doctors in the newborn unit paid out-of-pocket for medically-related costs of the very poor family.

**Table 5:** Direct cost categories and mean out-of-pocket expenditure by parents.

Cost category	Number of cases	Cost in US dollars mean ± SD* (median)
Medicines (not covered by insurance)	45	34.1 ± 25.6 (24.1)
Bedside diagnostics and therapeutics	56	18 ± 4.1 (19.2)
Hygiene supplies (the very poor family was exempt)	55	17.4 ± 3.6 (18)
Laboratory investigations not covered by insurance	17	17.4 ± 6.2 (17)
Other healthcare related costs (e.g. accommodation)	26	18 ± 9 (12.8)
Hospital bill (at referring hospital—out-born cases)	5	58 ± 45.6 (25.6)

*SD: standard deviation

Relatively richer families paid significantly higher costs out-of-pocket than poorer families. The OOP costs to families with a monthly income of over 300 dollars was significantly higher (p < 0001) than those who earned less (Table 6).

**Table 6:** Parental monthly income and mean out-of-pocket direct costs.

Parental monthly income category	Number of cases (families)	Total direct cost (SD)	Non-medical related costs (SD)
Less than 150	24	107 (57)	41.5 (38.3)
150–300	17	117 (51)	42.9 (31.2)
More than 300	8	161 (84)	81.9 (75.8)
No data	7	154 (125)	74.7 (86)

*SD: standard deviation.

Expenditure on transportation comprised 33.7% (IQR 19%– 50%) of direct costs and accounted for most non-medical related direct costs. Thirty one families had at least one other child at home, 71% (22/31) had someone looking after the children at home but only 9% (2/22) of families paid ($13/week) for childcare services. All but one of the fathers of newborns with perinatal asphyxia at RH visited every day of their newborn’s hospitalization; parents at DH visited less frequently but the LOS was shorter. More frequent parental visits was associated with higher non-medical related direct costs as shown in Table 4. There was no significant difference in the visiting pattern of fathers with preterm/LBW newborns at both hospitals.

**Fig 1:**
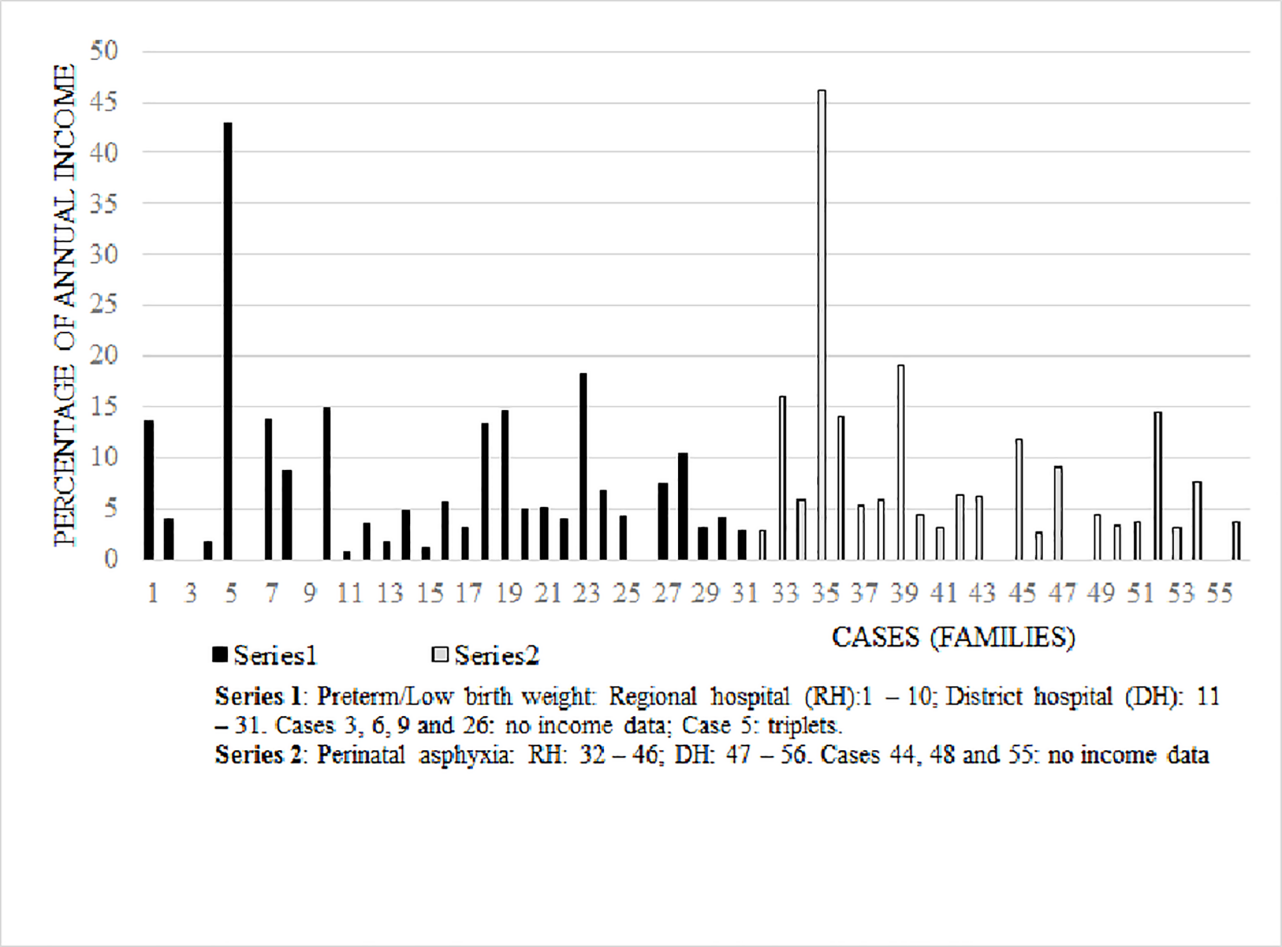
Proportion of annual income parents paid out-of-pocket for their hospitalized newborn.

All mothers of preterm/LBW practiced intermittent kangaroo mother care (KMC) but only 7 out of 31 mothers practiced continuous KMC in hospital. The cost ($6.4) of KMC kit at both hospitals was similar and mothers did not pay extra accommodation fee for the KMC ward. The proportion of combined parental annual income spent on inpatient neonatal care varied from 0.7% to 46.2% as shown in Fig 1. On average, families spent 8.1% and 9.1% of their annual income on acute care for preterm/LBW and perinatal asphyxia respectively.

### Indirect costs

Regarding productivity losses, 32 out of 42 fathers (Table 3) were in paid employment, 34% (11/32) reported lost earnings and 56% (18/32) took time off work (range: half day to 14 days) because of their child’s illness. Only one father had statutory paternity leave. Estimation of the actual financial loss fathers suffered as a result of their infant’s hospitalization was not ascertained as those in paid employment engaged in other productive activities with earnings that could not be verified.

The median time spent travelling to and from the hospital after the first day of hospitalization was 12 (IQR 4–26; range 1–180) hours. Overall, the median time fathers spent on medical-related hospital activities were: 60 (IQR 7–86; range 3–600) minutes waiting to see a health professional, 53 (IQR 21–75; range 4–595) minutes with a health professional, and 100 (IQR 30–190; range 15–1160) minutes on other hospital activities including organizing laboratory tests and buying drugs and other supplies.

Although both hospitals had reserved time (1 hour early morning and 1 hour in the evening) for fathers to visit their baby, most of that time was spent on hospital activities. Four fathers who reported zero time loss in hospital activities had preterm/LBW infants admitted at DH (mean length of stay 5 days (range 2–7 days); these fathers visited only once or twice during the newborn’s hospitalization. Parents of babies admitted at DH spent less time in hospital activities but only 29% (9/31) of these babies had laboratory tests as compared to 92% (23/25) of those at RH. Regarding loss of leisure time, 86% (48/56) of fathers reported disruptions in their leisure time.

## Discussion

We found that parents of hospitalised newborns in an urban setting in Ghana have significant financial constraints and opportunity costs at the point of care irrespective of their health insurance cover status. Limited resources and ineffective organization of service delivery undermined the quality of inpatient newborn care. Although the 2 hospitals delivered similar level of inpatient care, OOP expenditure of parents at RH was significantly higher than at DH. This was mostly due to the cost of oxygen therapy, longer LOS, higher use of medicines and therapeutics not covered by NHIS and higher transportation costs for families to RH. RH is in the middle of the city and DH is at the outskirts. The findings of this work confirms Lambon-Quayefio *et al* [17] postulation about overstretched health facilities and substandard neonatal services in urban settings in Ghana.

The study used TDABC micro-costing approach to identify important determinants of costs of neonatal services in the healthcare system and society. The health information system in Ghana like other LMIC precludes accurate assessment of costs of inpatient neonatal services from existing medical records. Micro-costing methods have been recommended for settings where routine systems are weak as disaggregation of costs is needed to better understand resource utilization in health service delivery [24]. We applied a comprehensive prospective approach to capture opportunity costs attributable to the illness and overcame limitations in routine documentation of clinical and financial data and other recognized constraints of conducting disease-specific cost analysis studies in LMIC [25–28]. The frontline health providers, the lead nurses who interviewed parents, described the experience as an “eye-opener” as they have not previously considered the costs borne by parents. Healthcare providers lack adequate understanding about the cost of services they provide and how these costs compare with health outcomes because costs are not usually linked to process of care and health outcomes [20].

The newborns in this study, especially those with perinatal asphyxia, have a high risk of long-term neuro-developmental morbidity [29]. On average parents spent 8% to 9% of their annual income for acute care, poorer families spent more, but families face significant economic burden in the future should their child develop significant impairment or disability from asphyxia or prematurity. Preventable newborn disorders pose significant long-term economic burden to families and society in LMIC [22, 30]. This study provides baseline cost data for future economic evaluations on neonatal health in Ghana.

### Health insurance package and out-of-pocket payments for hospitalised newborns

To ensure sustainability of NHIS, the Ghana-Diagnostic Related Grouping tariff system (G-DRG) was implemented in 2007 with FFS payment mechanism for medicines. G-DRG should cover direct health costs except medicines but we found FFS extended beyond medicines and included diagnostics (e.g. bedside random blood sugar), therapeutics (e.g. oxygen), ward hygiene supplies, and other related healthcare costs. In this study, direct medical-related costs accounted for about two thirds of OOP expenditure irrespective of health insurance status while in countries with more robust insurance schemes the major OOP expenditure by families with hospitalised newborns is transportation costs [22].

While the mean OOP expenditure on medical-related cost for perinatal asphyxia at RH and DH were $77.1 and $ 55.9 respectively, the NHIS reimbursement rate per patient was $77 and $54.4 respectively. For preterm/LBW, mean OOP expenditure was $140.8 at RH and $65.5 at DH while the reimbursement rate was $106.3 and $77.1 respectively. The reimbursement NHIS paid to hospitals was similar to the OOP financial expenditure parents with NHIS cover contributed for the health condition. NHIS reimbursement rates were based on assumptions of departmental resource utilization and not actual cost of procedures and resources utilized in the provision of care for a specific medical condition [20]. The hospitals provided basic in-patients infrastructural requirements, emergency supplies and general hygienic services with the low reimbursement they received on irregular basis. If higher reimbursement rates are given at regular predetermined periods to health facilities that provide specialized neonatal services it may improve the quality of care and reduce the economic burden of acute care on families. Irrespective of the relatively high financial burden on parents, NHIS provides significant financial relief to Ghanaian families compared to other countries in the sub-region where parents pay OOP for all services [31].

There was some variability in medical-related items parents purchased for the same medical condition at the 2 hospitals. We recommend a comprehensive review, costing and standardization of healthcare products and services relevant to inpatient neonatal care [32, 33]. Disease-specific standard lists and costs of products and services are useful for comparative economic evaluations within and between facilities [32]. Policy makers, hospital administrators and health insurance providers could use standard list to appraise health financing needs and the quality and value–outcomes achieved per dollar spent, of neonatal service [25, 33]. Standardization of healthcare products would equip potential parents with useful information to plan and reduce uncertainty and hardship when the unexpected happens.

### What costs do healthcare processes impose on parents?

The process of care at the study sites required patients or their family members to arrange the purchase of medicines and organize laboratory and radiological tests including collecting specimen bottles, sending specimens to the laboratory, and retrieving laboratory results. Most of these services are FFS so parents had to be present to pay but parents also served as porters for services covered by NHIS. Laboratory tests covered by NHIS were complete blood count and bilirubin measurements. Blood culture test was FFS. The uncertainty of daily healthcare needs for hospitalised newborns and the consequent unplanned expenditure on various healthcare inputs caused significant economic strain on parents and had negative effect on the quality of care provided. Some parents expended their resources on hygiene inputs and had no money for laboratory tests and medicines when their baby developed a health complication.

Most parents described hospital processes as “time consuming.” Inadequate directional signs at the hospitals may have contributed to the time parents spent in hospital activities and limited human resource could have contributed to the long waiting times before parents could discuss their baby’s condition with health professionals. The study demonstrates how cost analysis unmasks barriers to quality care and wastage of resources (human capital loss) due to inadequate financing and organization of healthcare services [34]. We did not monetize the cost of short-term parental productivity losses because fathers, including those in full employment, had other sources of income which we could not verify. Indirect cost were considered as opportunity costs.

The study findings reflect the true expenditure of families with hospitalised newborns at the study sites as families in Ghana do not receive medical benefits from the government and none of the families had private health insurance to mitigate unplanned health expenditure. Indeed, improved insurance coverage and financial protection for low income families may improve the quality of care and health outcomes and confer other benefits [35]. Although the short- and long- term human capital loss caused by birth asphyxia and preterm birth was not examined in this study the situation needs serious consideration because the long-term costs of poor quality neonatal services to society may overwhelm the health system of LMIC in the near future [7, 30].

### Limitations

The study has several limitations. Neonatal infections cause significant neonatal morbidity and mortality but it was excluded in this analysis because accurate diagnosis at enrolment was not feasible at the study sites. Secondly, micro-costing methods are more precise and accurate but they are less generalizable; however, this paper provides a detailed description of care processes and costing approach that may be useful to health facilities with similar circumstances as the study sites. Thirdly, the sample size of 56 families may be relatively small but overall, the study recruited over 10% of all eligible newborns despite the well-recognized rigour and expense of micro-costing studies and the strain it may have in settings without electronic medical records and limited human resource for direct observation studies. Even so, existing guidelines on economic evaluations do not provide specific recommendations for sample size in micro-costing studies [36].

Fourthly, self-reported cost data is prone to potential sources of bias. In this study every effort was made to minimize bias: the consenting process explained the importance of accurate data and parents knew they will not be reimbursed so there was no incentive to inflate prices. Also, receipts of purchases were examined, and the research team had good knowledge of the relative costs of goods and services in the city including transportation costs. Fifthly, maternal productivity losses and opportunity costs including loss of leisure time were not assessed because of the presumed traditional concepts about nursing mothers and paid maternity leave. This omission may have underestimated the financial and economic burden of neonatal illness to families. Sixthly, intangible costs including the psychological and emotional effects of newborn’s hospitalization on parents/family were not elicited as parents did not voluntarily disclose the information; this could have been due to limitations of the assessment tool or because parents were expended with the intensity of clinical activities. Intangible costs are complex to elicit but mothers are known to be more significantly affected.

## Conclusion

Cost analysis unmasks health system limitations that impact quality of care. TDABC is a rigorous relatively accurate approach that enables practitioners to examine healthcare processes and cost data and effectively redesign care to improve value for providers and patients. This work reveals important areas in the financing and organization of health services that have the potential to negatively impact the quality of care provided for hospitalised newborns in Ghana. The high out-of-pocket payments, limited para-clinical services, parental productivity losses, and parent-dependent hospital processes are vital areas that health facilities and the government should address. This study provides baseline data for further economic evaluations and useful information for reviewing the Ghana-DRG bundled payment reimbursement systems and the need for complementary health financing options for newborn services.

## Supporting information

S1 Appendix Parental costs for in-patient neonatal services in Ghana. (PDF)
