# Supplementary material for: Parental costs for in-patient neonatal services for perinatal asphyxia and low birth weight in Ghana
Source: PLoS One. 2018 Oct 12;13(10):e0204410. doi: 10.1371/journal.pone.0204410 (PMC6185862; doi:10.1371/journal.pone.0204410)
Supplement: S1 Appendix — (PDF) [file pone.0204410.s001.pdf]

S1 Appendix: Parental costs for in-patient neonatal services in Ghana

(Adapted from Thompson S, Wordsworth S. An annotated cost questionnaire for completion by patients. HERU Discussion Paper No. [03/01].)

PARENTS' QUESTIONNAIRE

Good assessment of the total cost (cost borne by health system and society) of providing quality care in newborn specialised or intensive care units (NICU) helps policy-makers and hospital managers understand the inputs needed to provide better care more efficiently. It also guides health insurance schemes to improve coverage for newborn services and reimburse hospitals appropriately. As part of the study which you have given consent for you and your baby to participate, this questionnaire seeks to document as much as possible all the costs your family is incurring during the process of your baby's admission. Please, we would encourage you to make every effort to answer all the questions accurately. All the information provided will be treated confidentially and you can contact the principal investigator (Christabel Enweronu-Laryea, 020 8154886) if you require further clarification.

Instruction: **Please fill in the appropriate answer (indicate number) for each parent**

1.0 SOCIODEMOGRAPHICS

Baby's Study ID: ____ Baby's initials _________

		**Father**	**Mother**
**1.1**	What is your relationship to the baby?		
**1.2**	What is your marital status?<br>Single.........1 Married or living as married....2 Separated ...3<br>Divorced....4 Widowed.....5 Other (specify)........................6		
**1.3**	What is the highest level of education you completed?<br>Primary......1 Some secondary........2 All secondary.......3<br>College......4 University......5 Post-graduate university…..6<br>None………...7		
**1.4**	What is your employment status?<br>Full-time work.......1 Part-time work.......2 Retired........4<br>Homemaker...........3 Currently seeking work...........5<br>Other (specify)................6		
**1.5**	Please provide an estimate of your **monthly** income (Ghana cedis) from all sources. Please write the appropriate range in the box to indicate the range within which each parent's income belongs.<br>< 250 | 750 - 1000 | 3000 - 4000<br>250 - 500 | 1000 - 2000 | 4000 - 5000<br>500 - 750 | 2000 - 3000 | > 5000		
**1.6**	How many adults are there in your household? Please write number		
**1.7**	How many children are there in your household? Please write number		

2.0 TRANSPORTATION

This part of the questionnaire asks about costs of travelling to the hospital because of your baby.

Method of transportation <u>on the day of hospital admission</u> (for both parents and the newborn)

		**Father**	**Mother**
**2.1**	Please indicate the number that best describes how you travelled from your home to the hospital. If you used more than one form of transport please indicate the way you travelled for the **MAIN** (longest in terms of distance) part of your journey.		
**2.2**	Please indicate **any other forms** of transport you used to travel from your home to the hospital to because of your baby. You may circle more than one answer if appropriate. Please circle 'No other forms of transport' if you used only one form of transport as indicated above.		
	Bus/Trotro ....1 Commercial motorbike………….. 2<br>Taxi............................ 3 Private vehicle................................4<br>Bicycle.........................5 Walked...7<br>Ambulance service…...8 Train..6<br>No other forms of transport …..........9		

Cost of transportation (Ghana cedis) <u>on day of hospital admission</u> (for both parents)

		**Father**	**Mother**
**2.3**	If you travelled by **public transport (e.g. bus, taxi, train)** for the **MAIN** (longest in terms of distance) part or the entire journey, please write the cost in the space provided. If you did not travel by public transport or you did not pay a fare write zero		
**2.4**	If you travelled by **commercial motorbike** for part of the journey please write the cost in the space provided. Put zero if you did not travel by taxi at all or if you did not pay a fare.		
**2.5**	If you travelled by **taxi** for part of the journey please write the cost in the space provided. Put zero if you did not travel by taxi you did not pay a fare.		
**2.6**	If you travelled by **private** car/motorbike for part or the entire journey how many kilometres did you travel one-way? Please write the number of kilometres or the route of your journey below. Put zero if you did not travel by private car or motorbike at all. Route ………………………………… ………………………………………………………………………………		
**2.7**	If you travelled by **private** car/motorbike for part or all of the journey and had to pay **tolls or parking fees** how much did these amount to? Please write the cost below in the space provided. Put zero if you did not travel by private car or motorbike at all or did not pay tolls or parking fees.		

2.8 How long did it take to travel from your home or office (circle as appropriate) to the ward to visit your baby? Please write the number of hours and minutes below.

Hours_____________ minutes___________________

2.9 Will you return home using the same form of transport? **Yes……...1** **No………2**

If NO please specify: Mode of transportation______________________________

Expected duration of return journey___________________

Expected cost or mileage of return journey______________

Can confirm these if parent returns to visit the <u>next day</u>

3.0 CHILD CARE AND OTHER DEPENDENTS' COST – *information from mother, but both parents should provide information if they are not living together*

Interviewer: *This section should not be filled on the day of admission as parents need a few days to organize their home circumstances*

This section asks about any assistance that you need to look after your child/children or other dependants when you are at the hospital visiting your baby in NICU. We are interested only in assistance that you need because of your baby's admission in NICU and not for any other reason.

3.1 Do you have other children or dependents that need your care at home?

Yes…………………… 1   No (if no go to section 4) ………………2

3.2 Did you get someone to look after your child/children or other dependants because of your baby's admission to NICU?

Yes................................1   No (go to section 4) …………..............2

3.3 How many hours and minutes would the caregiver spend looking after your child/children or dependants per day?

Hours_________   minutes_________

3.4 Concerning the **main caregiver**, that is the person who is mostly responsible for looking after your child/children/dependants while you baby is in NICU - did you pay that person?

Yes................................1   No (go to question 3.6)............................2

3.5 How much would you pay the **main caregiver** to look after your child/children/dependants while your baby is on admission at NICU? Please specify the amount and circle as appropriate if per day or per week.

Cedis______________   pesewas__________________   per day / per week

3.6 If the **main caregiver** was not paid, what would that person have been doing as their main activity if they had not been looking after your child/children/dependants while your baby is at NICU? Please circle the number that best describes the person's main activity.

Housework................ 1   Childcare.................. 2   Voluntary work.................3

Leisure activities........ 4   Seeking work……… 5   Paternity leave…………..6

On sick leave.............7   Paid work................. 8   Attending school 9

Caring for a relative or friend.................10   Other (please specify)……................... 11

3.7 If the **main caregiver** took time off from paid work or business activity if self-employed to look after your child/children/dependants while your baby is at NICU approximately how much time did they take off paid work? Please write the number below.

Days________________   Hours_____________   Minutes_____________

3.8 What is the caregiver's main occupation? ____

4.0 PARENTAL TIME COSTS AND PRODUCTIVITY LOSSES – *fathers only*

This section asks about effect of your baby's admission to NICU on your paid work, unpaid work or leisure activity in the previous 24 hours or since the last time you came to visit your baby in the hospital.
Paid work is any work you do for which you receive money payment, includes self-employed work.
Unpaid work means activities such as household chores, shopping, caring for children, voluntary work, and non-leisure activities that involve your time but for which you do not get paid.
Leisure activity includes any activity in which you engage that is not related either to paid or unpaid work, for example, hobbies, pastimes and social activities.

4.1 What is your main occupation/job? ____

How many hours do you work per day? ____________________
How many days do you work per week? ____________________

4.2 Before your baby's hospital admission, were you in paid work?

Yes …………1 No (please go to question 4.8) 2

4.3 How many hours on average do you do paid work? Please indicate below.

Number of **hours** per day________ Number of **days** per week ____________

4.4 Does your paid work /business activity make provisions for paid paternity leave?

Yes ……….........1 No (please continue from question 4.6) 2

4.5 If your work provides paid paternity leave, please indicate how many days of paid leave you are entitled to.

Number of days of paid paternity leave_____________

4.6 If you took time off from paid work because of your baby's admission approximately how much time have you taken off work/business activity? Please indicate how much time you took off work.

Days_____________ Hours____________ Minutes____________

4.7 Will you lose earnings/money as a result of taking time off paid work?

Yes……………….....1 No…………………………..2

4.8 The first time you visited your baby in NICU, how long did you spend there? Please include in your answer the time spent waiting to see your baby or a health worker, time spent with the doctor or nurse, time spent on other hospital activities for your baby e.g. registration, laboratory services etc.

Waiting time: Hours_________ minutes_________
Time with doctor or nurse: Hours_________ minutes_________
Time spent with your baby: Hours_________ minutes_________
Time spent on other hospital activities: Hours_________ minutes_________

4.9 What would you otherwise have been doing as your **MAIN** activity if your baby was not in NICU?

Paid work/business.......... 1 Housework..................4 Childcare.............. 7
Leisure activities............. 2 Voluntary work.............5 Seeking work……..8
Attending school .... 3 Paternity leave……….6 Sick leave..............9
Caring for a relative or friend.................10 Other (specify)…………………….... 11

4.9.1 Before your baby's hospital admission, how many hours a week, on average, did you spend on unpaid work (e.g. household chores, shopping, caring for children or voluntary work)? Please write the number of hours below.

Number of unpaid work: hours per day_________ days per week__________

Parental time costs and productivity losses – *cont'd*

Please complete the table daily.

Indicate the time you spend each day of your baby's admission in NICU waiting to speak to a healthcare worker about your baby, discussing the care of your baby with a healthcare worker, or going to the laboratory to get bottles for blood test or collect test results or going to the pharmacy in the hospital or elsewhere to buy medicines required for your baby. Please only include times that were specifically related to the care of your baby.

Parental time and activity in healthcare								
Period during NICU admission	Date of baby's hospital admission:							
	Waiting time to see doctor/nurse		Time spent with nurse or doctor about your baby		Time spent going to & waiting at pharmacy or laboratory etc.		**Total**	
	Hour	Minute	Hour	Minute	Hour	Minute	Hour	Minute
Week 1 (write day/date)								
Day 1								
Day 2								
Day 3								
Day 4								
Day 5								
Day 6								
Day 7								
Week 2								
Day 8								
Day 9								
Day 10								
Day 11								
Day 12								
Day 13								
Day 14								
Week 3								
Day 15								
Day 16								
Day 17								
Day 18								
Day 19								
Day 20								
Day 21								
Week 4								
Day 22								
Day 23								
Day 24								
Day 25								
Day 26								
Day 27								
Day 28								

Transportation TO HOSPITAL during course of your baby's admission								
Date of admission:								
Period during NICU admission	Method of transportation		Duration of journey		Cost of transportation in cedis and pesewas		Private vehicle	
	MAIN	Other form	Hour	Minute	MAIN	Other form	Km or Route	T & P
Week 1 (write day/date)								
Day 1								
Day 2								
Day 3								
Day 4								
Day 5								
Day 6								
Day 7								
Week 2								
Day 8								
Day 9								
Day 10								
Day 11								
Day 12								
Day 13								
Day 14								
Week 3								
Day 15								
Day 16								
Day 17								
Day 18								
Day 19								
Day 20								
Day 21								
Week 4								
Day 22								
Day 23								
Day 24								
Day 25								
Day 26								
Day 27								
Day 28								

MAIN form of transportation is the method used for the longest distance of the journey

Any other forms of transportation used but different from the **MAIN**

Code: Bus/Trotro ....1 Private vehicle............4 Walked........................7
Commercial motorbike…... 2 Bicycle........................5 Ambulance service…...8
Taxi.................................... 3 Train...........................6 Other (specify)…..........9

If private vehicle (bicycle, motorbike, car/van) transportation write cost of toll and parking fee in the T&P column. Document mileage (km) if possible - for private vehicle transport only.

Transportation FROM HOSPITAL to home/office during course of your baby's admission									
Date of admission:									
Period during NICU admission	Method of transportation		Duration of journey		Cost of transportation in cedis and pesewas		Private vehicle	
	MAIN	Other form	Hour	Minute	MAIN	Other form	Km or Route	T & P
Week 1 (write day/date)								
Day 1								
Day 2								
Day 3								
Day 4								
Day 5								
Day 6								
Day 7								
Week 2								
Day 8								
Day 9								
Day 10								
Day 11								
Day 12								
Day 13								
Day 14								
Week 3								
Day 15								
Day 16								
Day 17								
Day 18								
Day 19								
Day 20								
Day 21								
Week 4								
Day 22								
Day 23								
Day 24								
Day 25								
Day 26								
Day 27								
Day 28								

MAIN form of transportation is the method used for the longest distance of the journey

Any **other forms** of transportation used but different from the **MAIN**

Code: Bus/Trotro ....1 Private vehicle............4 Walked.........................7
Commercial motorbike…... 2 Bicycle........................5 Ambulance service…...8
Taxi.................................... 3 Train...........................6 Other (specify)…...........9

If private vehicle (bicycle, motorbike, car/van) transportation write cost of toll and parking fee in the T&P column. Document mileage (km) if possible - for private vehicle transport only.

5.0 MEDICINES and INFUSIONS

This section asks about any prescribed medications (all forms of drugs and intravenous fluids) that **<u>you have been asked to buy for your baby during this admission</u>**. Please show the prescription and also show the receipt you were given at the time you purchased the medicine to the interviewer.

	Period of NICU admission (date)	Medicine prescribed	Medicine given to baby	Cost (cedis)	Comment<br>**Y** – receipt seen<br>**N** – receipt not seen<br>**X** – by insurance
1					
2					
3					
4					
5					
6					
7					
8					
9					
10					
11					
12					
13					
14					
15					
16					
17					
18					
19					
20					
21					
22					
23					
24					
25					
26					
27					
28					
29					
30					
31					
32					

6.0 LABORATORY TESTS AND RADIOLOGICAL INVESTIGATIONS

This section asks about any laboratory tests (e.g. blood tests) or radiology investigations (e.g. x-ray) that you have been asked to pay for your baby during this admission. Please show the prescription and also show the receipt you were given for the test or service to the interviewer.

	Period of NICU admission (date)	Laboratory or radiological investigation prescribed	Laboratory or radiological investigation done	Cost (cedis)	Comment<br>**Y** – receipt seen<br>**N** – receipt not seen<br>**X** – by insurance
1					
2					
3					
4					
5					
6					
7					
8					
9					
10					
11					
12					
13					
14					
15					
16					
17					
18					
19					
20					
21					
22					
23					
24					
25					
26					
27					
28					
29					
30					
31					
32					

8.0 SUPPLIES: Medical and Miscellaneous

This section asks about any medical supplies (e.g. formula feed, cannula, feeding tube, linen, diaper, plaster, feeding cup, wipes, cotton wool, providone iodine, savlon, soap, towel, KMC wrap, etc) that you have been asked to pay for or buy for the care of your baby during this admission. Please show the prescription and also show the receipt you were given for the purchase or service to the interviewer.

	Period of NICU admission (date)	Supplies recommended	Tick if supplies provided by parents - indicate quantity	Cost (cedis)	Comment<br>**Y** – receipt seen<br>**N** – receipt not seen<br>**X** – by insurance
1		Cot sheet			
2		Baby clothes			
3		Baby wipes			
4		Towel			
5		Diaper			
6		Oil/Vaseline			
7		Savlon			
8		Soap			
9		Baby socks & cap			
10		Tissue paper			
11		Methylated spirit			
12		Cotton wool			
13		Pail for baby wash			
14		Feeding cup			
15		Bag for baby items			
16					
17					
18					
19					
20					
21					
22					
23					
24					
25					
26					
27					
28					
29					
30					
31					
32					
33					
34					
35					
36					

9.0 ANY OTHER COSTS – ADDITIONAL INFORMATION

This section asks whether there are any other costs you have incurred as a result of your baby's hospital admission which has not been addressed in this questionnaire. These may include e.g. cost of accommodation, telephone calls, or any other additional expenses **directly connected** to your baby's admission in hospital.

9.1 Have you incurred any other costs because of your baby's hospital admission?

Yes 1

No 2

9.2 If yes, what were they for and how much did you spend? Please provide more information below:

Activity / Item	What was it for?	How much did it cost? (cedis)

9.3 Is there anything else that you would like to tell us about your costs (time, money, family life, etc) during your baby's hospital admission?

……………………………………………………………………………………………………
……………………………………………………………………………………………………
………..……………………………………………………………………………………..……
……………………………………………………………………………………………………
……………………………………………………………………………………………………
……………………………………………………………………………………………………
…………………………………………………………………..…………………………..……
……………………………………………………………………………………………………

________________________________THANK YOU________________________________
